# Impact of Water Pollution on Trophic Transfer of Fatty Acids in Fish, Microalgae, and Zoobenthos in the Food Web of a Freshwater Ecosystem

**DOI:** 10.3390/biom9060231

**Published:** 2019-06-14

**Authors:** Shahid Mahboob, Khalid Abdullah Al-Ghanim, Fahad Al-Misned, Tehniat Shahid, Salma Sultana, Tayyaba Sultan, Bilal Hussain, Zubair Ahmed

**Affiliations:** 1 Department of Zoology, College of Science, King Saud University, Riyadh 11451, Saudi Arabia; kghanim@ksu.edu.sa (K.A.A.-G.); almisned@ksu.edu.com (F.A.-M.); zahmed@ksu.edu.sa (Z.A.); 2 Department of Zoology, Government College University, Faisalabad-38000, Pakistan; sal545@live.com (S.S.); arif123@yahoo.com (T.S.); prof_bilal@yahoo.com (B.H.); 3 House No. 423, Block M-1, Street No. 14, Lake city, Lahore 55150, Pakistan; tehniatshahid123@gmail.com

**Keywords:** Fatty acid, fish, food web, periphyton, trophic transfer, water pollution, zoobenthos

## Abstract

This research work was carried out to determine the effects of water contamination on the fatty acid (FA) profile of periphyton, zoobenthos, two Chinese carps and a common carp (*Hypophthalmichthys molitrix, Ctenopharygodon idella* and *Cyprinus carpio*), captured from highly polluted (HP), less polluted (LP), and non-polluted (NP) sites of the Indus river. We found that the concentration of heavy metals in the river water from the polluted locations exceeded the permissible limits suggested by the World Health Organization (WHO) and the US Environmental Protection Agency (EPA). Fatty acid profiles in periphyton, zoobenthos, *H. molitrix*, *C. idella*, and *C. carpio* in the food web of river ecosystems with different pollution levels were assessed. Lauric acid and arachidic acids were not detected in the biomass of periphyton and zoobenthos from HP and LP sites compared to NP sites. Alpha-linolenic acid (ALA), eicosadienoic acid and docosapentaenoic acid were not recorded in the biomass samples of periphyton and zoobenthos in both HP and LP sites. Caprylic acid, lauric acid, and arachidic acid were not found in *H. molitrix*, *C. idella*, and *C. carpio* captured from HP. In this study, 6 and 9 omega series FAs were identified in the muscle samples of *H. molitrix*, *C. idella* and *C. carpio* captured from HP and LP sites compared to NP sites, respectively. Less polyunsaturated fatty acids were observed in the muscle samples of *H. molitrix*, *C. idella*, and *C. carpio* collected from HP than from LP. The heavy metals showed significant negative correlations with the total FAs in periphyton, zoobenthos, and fish samples.

## 1. Introduction

The aquatic fauna and flora of river ecosystems comprise a complex assemblage of different communities and are biologically important because of the interlinking between different trophic levels. These aquatic food chains are very feeble and sensitive to contaminants, especially to the toxicity of exogenous chemicals and heavy metals that are discharged into freshwater reservoirs due to various human activities. Heterotrophic aquatic organisms in food chains consume a variety of metalloids and xenobiotic compounds, which usually cause immutable degradation of the planktonic life at higher concentrations [1,2]. The toxic response in freshwater fish species to contaminated environments has been reported on a global scale [3,4]. The uptake of heavy metals into the aquatic food chain can occur either by dietary or non-dietary routes [5]. Therefore, the concentration of heavy metals in fish normally indicates levels present in sediment and water that is specifically in freshwater reservoirs where the fish is captured from [6], as well as the time of exposure [7]. The concentration of essential metals, if increased above the normal metabolic needs of fish, may become toxic for the fish and for the ultimate consumer, humans [8]. Heavy metals may accumulate in primary producers such as microalgae, where diatoms ultimately transfer them to other trophic levels [9]. Heavy metals are ingested by fish and bio accumulate in the liver, kidneys, and other vital organs through adsorption and absorption [7].

Lipids are considered to be one of the most essential nutrients, which affect the growth and development of fish and other organisms [10], and alleviate immune competence [11]. Essential lipids are nutritionally important for the consumers in the food chain because they promote the growth and development and overall health of aquatic fauna and flora of aquatic communities in freshwater ecosystems [12,13,14]. Kainz et al. [15] proposed that the trophic movement of fatty acid (FA) in the food chain may be used as a physiological biomarker for monitoring the status of contamination in freshwater ecosystems. Kainz et al. [15] further mentioned that this trophic movement of FA in the food chain may be used as a physiological biomarker for observation of the status of contamination in freshwater ecosystems. Thus, it is necessary to ensure the abundance of microalgae and zoobenthos for trophic transfer into higher levels in the food web to ensure the transfer of FA and polyunsaturated fatty acid (PUFA) to the fish [16]. Currently, there are no comprehensive reports in the literature describing the interlinking trophic movements of PUFA and the impact of water pollution in the river ecosystems. The latter is still poorly understood with reference to FA profiles of periphyton, zoobenthos, and fish, and effect of contaminants. periphyton and zoobenthos can be used as valuable indicators to determine the effect of contamination and the synthesis of FA in freshwater ecosystems [17].

Aquaculture plays an important role in providing good quality animal protein and provides sustainable livelihood opportunities and food security for the ever increasing world population [18,19]. Fish are recognized as an important part of the human diet, owing to its balanced ratio proteins/PUFAs, including omega series FAs [20] which may reduce the risk of heart diseases. Because of the nutritional and pharmaceutical importance of PUFAs, researchers in the discipline of fishery sciences have been paying them increasing attention [21,22].

The bioaccumulation of metals in fish is triggered by the accumulation of these elements in phyto- and zooplankton; however, this event ceases to be the most relevant as long as biomagnification takes place. Biomagnification can have serious impacts on the food chain [23]. Fish has the potential to accumulate more metals from food and water [24]. Kainz and Fisk [25] mentioned that most of the FAs and pollutants move trophically through the food chain, ultimately having effects on the final consumer. This situation warrants an understanding of the fate of FAs and the impact of heavy metals contamination on the variability of FA on the food chain in river ecosystems. Variation in FAs dynamics in the food web is linked to increases in environmental stress and habitat destruction due to water pollution within a freshwater ecosystem [26]. Moreover, the disparity in FAs and pollutant trophic movement in the food chain may give insights into ecological functions and their impact on habitat and environmental stress. Keeping this in mind, it is necessary to investigate the interlink and biotransformation of FAs in the food web and the relationship with water pollution. This requires assessing and contrasting the trophic movement of lipids and pollutants in the aquatic food chain. The main aim of research was (i) to assess the fatty acid profiles in periphyton, zoobenthos, *Hyphpthalmichthys molitrix*, *Ctenopharyngodon idella*, and *Cyprinus carpio* in the food web of river ecosystems with different pollution levels; (ii) to assess their flow in aquatic ecosystems; (iii) to explore their potential for evaluating and monitoring the health of aquatic habitats; (iv) and to apply FA profiles as a possible physical biomarker of environmental stress from heavy metal pollution.

## 2. Materials and Methods

### 2.1. Study Area

The Indus river is the longest river in Pakistan. The Indus River originates on the Tibetan Plateau, enters into towards Gilgit-Baltistan from Ladakh, and then flows from Punjab Province and joins into the Arabian Sea. It is the largest river in Pakistan with a total catchment basin of about 1,165,000 km^2^ (450,000 m^2^) https://en.wikipedia.org/wiki/Indus_River).

**Figure 1:**
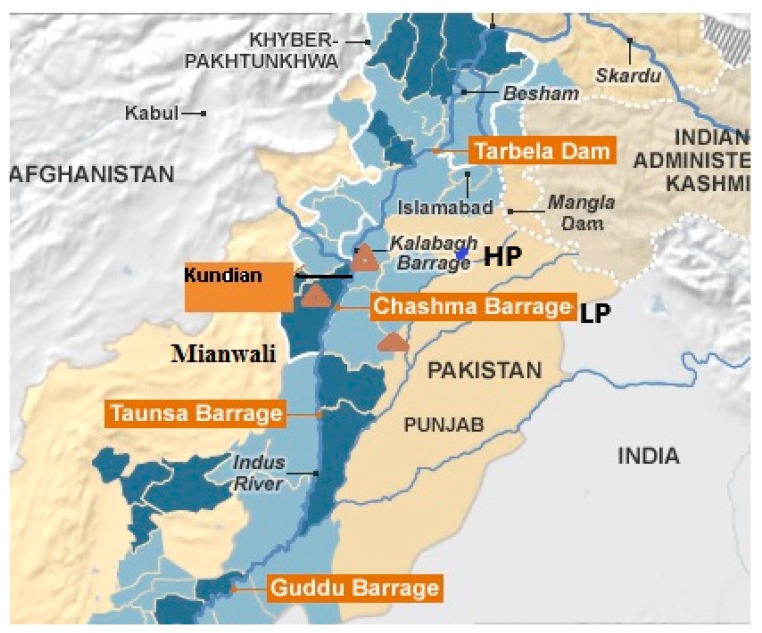
Map of the locations of sampling sites on the Indus River (Kundian Barrage, Kalabagh Barrage, and Chashma Barrage) [25]. Source: OCHA (United Nation Office for the Coordination Humanitarian Affairs).

The Mianwali District is situated in the province of the Punjab and is about 200 m above sea level (Figure 1; 25). The Mianwali is one of the districts in the province of the Punjab and is about 200 m above sea level [27]. This district is rich in minerals, clay, coal, gypsum, limestone, etc., which are excavated for commercial purposes. In this district there is also a nuclear power plant and the Chashma Hydel power plant, which are adding electricity into the national electricity grid. The temperature ranged between –2 °C and 51 °C with 255 mm of rainfall [28]. The experimental sites were selected in Area 1 (Kalabagh; high pollution (HP) site), Area 2 (Chashma; low pollution (LP) site), and non-polluted (NP) site (Area 3; Attock) along the River, and these sites were 35 km apart from each other.

### 2.2. Collection and Preparation of Fish Samples

*Hypophthalmichthys molitrix* is planktivorous and consumes the organisms within lower multiple lower trophic levels across a range of habitats. Grass carp (*Ctenopharyngodon idella*) is a large cyprinid and is a voracious feeder. Small grass carp consume planktonic crustaceans, rotifers, and insect larvae, while the adults are completely vegetarian. *Cyprinus carpio* is a popular benthivorous fish that has larger bottom–up effects than other benthivorous fish. The bottom–up effects of *C. carpio* mainly depend on the incorporation of benthos-derived nutrients and the release of nutrients from bottom sediment during grazing on benthos. Twenty-one specimens of *H. molitrix, C. idella*, and *C. carpio* each were captured from HP, LP and NP sites for an evaluation of the fatty acid profiles. A total of 63 fish specimens were procured with the help of fishermen. The average weight ranged from 900 to 1200 g. Fish specimens were transferred live in polyethylene bags to the laboratory. Muscle samples were processed as per the method mentioned by reference [29]. This study was approved by the Ethics and Animal Welfare Committee of the Department of Zoology, GC University, Faisalabad (Ethical code number: GCUF/Zool/EAWC/34).

### 2.3. Analysis of Water Samples

Water samples were collected in hydrographic bottles of 32 oz capacity at the depth of 30 cm below the surface from the three determined sampling sites for the determination of selected physiochemical parameters and selected heavy metals through an atomic absorption spectrophotometer (“Hitachi polarized Zeeman AAS, 2000 series”) by following the procedure as mentioned by reference [30]. The water samples were collected in the morning and these were stored in iceboxes before being taken to the laboratory for analysis. Different dilutions of Hg, Sn, Cr, Pb, Zn, Mn, Cu, and Cd were made to check the accurateness of the equipment during the analysis of samples. The quality control and quality assurance protocol was followed as mentioned in our previous published work [9]. Calibration curves were plotted and validated with their corresponding R^2^ values for the detection of each metal. The values of R^2^ of the curves were 0.99983, 0.99981, 0.99951, 0.99984, 0.99926, 0.99987, and 0.99982 for Hg, Sn, Cr, Pb, Zn, Mn, Cu, and Cd, respectively.

### 2.4. Periphyton Sampling

Periphyton samples were obtained from the three experimental locations by following the methodology of references [28,29]. “A 10 × 10 cm steel frame was fixed at the bottom at three points of each location and composite them to collect the periphyton, then the pebble was removed. The periphyton samples was cleaned from the pebble surface using brushes, after which it was washed with river water. Aliquots from this volume were centrifuged at 2500 g for 15 min for the further analysis of metals and fatty acids” [31].

### 2.5. Zoobenthos Sampling

Zoobenthos samples were obtained from the experimental locations at three points and composite them through a Samples Surber-type kick-bottom sampler as mentioned by reference [31].

### 2.6. Fatty Acid Profiling

The lipid components were obtained from the fish muscle, periphyton, and zoobenthos samples with help of Soxhlet extractor (Electrothermal EME6 England), as described by reference [9]. “The extracted lipids were transformed to fatty acid methyl esters using methanolic sulfuric acid by an esterification procedure”, as described by references [32,33]. The fatty acid profiling was carried out by following the methods of reference [34], through gas chromatograph (Perkin Elmer Model 3920) with flame ionization detector (FID) column 2 m in length and 2 mm in diameter. The chromatograms recorded from all samples were used to observe the retention time of each fatty acid (Fatty acid methyl esters (FAMEs)) and these were compared to the chromatogram of a standard (mixture of pure FAMEs) as described by reference [35].

### 2.7. Statistical Analysis

The data obtained was processed using Minitab software for analysis of variance (ANOVA) to assess the dissimilarity between various parameters of this study between the three sampling sites. Duncan’s multiple range test (DMR test) (*p* < 0.05) was used to compare the means. “Shapiro-Wilk’s W test and Levene’s test” was used for normality and homogeneity of the data when necessary [36]. Correlation coefficients were calculated to determine the relationship between the concentration of heavy metals and the total FAs profile in fish and planktonic life from three sampling sites.

## 3. Results

### 3.1. Physico-Chemical Factors and Heavy Metals

The physico-chemical parameters of the water samples from sampling sites (HP, LP and NP) are presented in Table 1. The level of salinity of HP was about 2%, found to be close to the level of salinity of the open ocean (normally about 3%). pH levels were 12.1 ± 0.36, 8.6 ± 0.12, and 8.1 ± 0.08 in HP, LP, and NP sites, respectively. The pH level was very high at the HP site. The highest biochemical oxygen demand (81.2 ± 1.10 mg/L) and chemical oxygen demand (195.8 ± 1.16 mg/L) were recorded at the HP site. The concentration of total dissolved solids (2445.5 ± 8.41 mg/L) and total suspended solids (329.6 ± 6.41 mg/L) were very high at the HP site. The concentration of phenols and sulfates were highest at the HP site, closely followed by the LP site. The level of phenols at the HP site was 15 times higher than at the NP site (Table 1).

The concentration of studied heavy metals are presented in Table 1. These concentrations exhibited significant variations between the three sites. The level of Sn, Cr, Pb, Mn, Cu, Cd, and Hg at the HP in fish muscle, periphyton, and zoobenthos biomass were highest at HP compared to LP and NP site and was above the upper limits stated by reference [34] (Table 1 and Table 2). The highest level of Cu in muscle samples was detected in *C. carpio* and plankton from HP, followed by LP sites. The maximum level of metals was recorded in the muscle samples of *C. carpio* captured from HP, followed by LP and NP sites. The lowest concentration of these metals was recorded in the muscles of *C. idella* (Table 2).

### 3.2. Fatty Acids Profile

The saturated fatty acids (SFAs) were low in the biomass of periphyton and zoobenthos obtained from HP and LP sites, compared to NP sites (Table 3). Lauric acid and arachidic acids were not detected in the biomass of periphyton and zoobenthos from HP and LP. The Environmental Protection Agency (EPA) value was significantly higher in the biomass sampled from NP, compared to HP and LP. The number of monounsaturated fatty acids (MUFAs) was higher in samples of periphyton and zoobenthos from NP (Table 3). Palmitoleic acid, vaccenic acid, oleic acid, eicosenic acid and erucic acid were not detected in periphyton biomass samples from HP and LP. PUFAs level was greater in periphyton and zoobenthos biomass from NP, compared to HP and LP sites. Alpha-linolenic acid (ALA), eicosadienoic acid, docosapentaenoic acid and docosapentaenoic acid were not detected in the biomass of periphyton and zoobenthos sampled from HP and LP. The percentage of EPA and DHA were higher in the periphyton biomass from HP, compared to NP (Table 3).

The fish captured from HP exhibited lower FAs and SFAs compared to the fish captured from LP (Table 4). The percentage of PUFAs in *H. molitrix, C. idella*, and *C. carpio* captured from HP was 32.32 ± 0.65, 7.19 ± 0.35, and 26.13 ± 0.82%, respectively. The percentage of PUFAs in *H. molitrix, C. idella*, and *C. carpio* captured from NP was 48.65 ± 1.11, 41.55 ± 0.97, and 44.15 ± 1.90%, respectively. The percentage of MUFAs and SFAs in *H. molitrix, C. idella*, and *C. carpio* captured from HP were 6.74 ± 0.29, 5.14 ± 0.17, and 5.46 ± 0.54 and 43.38 ± 2.45, 62.94 ± 3.05, and 74.07 ± 4.14%, respectively. The total MUFAs and SFA profiles in *H. molitrix, C. idella*, and *C. carpio*, captured from LP showed a similar trend of fluctuations to fish from HP (Table 4).

The maximum percentage of SFAs in *C. carpio* was observed in HP. A decrease in the abundance of *C. carpio* was noticed during the study period (Table 4). Caprylic acid (C8:0), lauric acid (C12:0) and C20:0 arachidic acid were not found in *H. molitrix, C. idella*, and *C. carpio* from HP. A very small amount of lauric acid (C12:0) and C20:0 arachidic acid was recorded in *H. molitrix, C. idella*, and *C. carpio* from LP. Eicosapentaenoic acid was not detected in any of the fish species collected from HP sites. In this study, 6 and 9 omega series FAs were found in muscle samples of *H. molitrix, C. idella*, and *C. carpio* from HP and LP, respectively. Linoleic acid (C18:4(n-3), eicosadienoic acid (C20:2 (n-6), and docosapentaenoic acid (C22:4 (n-6) were not recorded in fish from HP. Eicosapentaenoic acid (C20:5 (n-3) was detected only in the muscle samples of *H. molitrix* from HP. Total 11 omega series FA were recorded in muscle of *H. molitrix, C. idella*, and *C. carpio* from NP sites. Caprylic acid was not detected in *H. molitrix, C. idella*, and *C. carpio* from LP sites (Table 4). Myristic acid (C14:0) was recorded as 0.44 ± 0.05, 1.826 ± 0.21, and 2.651 ± 0.22% in *H. molitrix, C. idella*, and *C. carpio*, respectively, from HP. Myristic acid was determined as 1.54 ± 0.04, 1.37 ± 0.03, and 0.53 ± 0.02 and 0.14 ± 0.01, 4.68 ± 0.55, and 7.79 ± 0.44% in *H. molitrix, C. idella*, and *C. carpio* from LP and NP sites, respectively. Arachidic acid was not found in *H. molitrix* and *C. carpio* from HP sites. Oleic acid (C18:1 (n-9) was not detected in *C. idella* collected from HP sites. C16:1 (n-7) (palmitoleic acid), C16:1 (n-9) (Cis-7 hexadecenoic acid), and C20:1 (N-9) (Eicosenoic acid) were not found in these fish species captured from HP. C18:1 (n-7) (cis-vaccenic acid) was detected only in the muscle sample of *H. molitrix* from HP. C16:1 (n-7) was detected only in the muscle samples of *H. molitrix* from LP sites. The concentration of C16:1 (n-7) was only determined as 0.72 ± 0.04 in *C. carpio* from NP sites (Table 4).

Correlation indices that were calculated among the concentrations of total FAs in periphyton, zoobenthos, fish muscle, and heavy metals in water samples are presented in Table 5. It has been observed that Sn, Cr, Pb, Zn, Mn, Cu, Cd, and Hg indicated significantly negative correlations with total FA profile in periphyton, zoobenthos, and fish samples from HP and LP (Table 5). Highly significant negative correlations were observed among Cr, Zn, Mn, and Cu and the total fatty acid profile samples of periphyton, zoobenthos, and fish collected from the HP site. The variation in FA found positively correlated with the level of contamination of these heavy metals in the food web. The health of the aquatic system was found to be significantly affected by the water quality of the HP and LP sites of the river compared to the NP site, which possibly causes decreases in the abundance of periphyton and fish populations in the aquatic system.

## 4. Discussion

The trophic transfer of FAs from periphyton to the organisms at higher trophic levels is important for their health and growth [14,37]. This movement of important nutrients in the food chain may be affected by different contaminants in the freshwater ecosystem [15]. The metals and metalloids, phenols, and organic contaminants in freshwater ecosystems enter the food of aquatic animals from various sources, including anthropogenic activities, and accumulate in planktonic life and fish. The heavy metals which accumulate can cause physiological stress on FA at different trophic levels in the food chain, and ultimately in humans [16,37].

Fish are used as a bioindicator for different organic and inorganic pollutants in freshwater ecosystems due to their presence in different trophic levels, because of their age, size, and mode of nutrition [15]. Various factors have effects on the distribution of aquatic fauna and flora in freshwater reservoirs [38,39]. Abiotic parameters are considered to mostly affect the pattern of distribution and richness of planktonic life [39,40]. The metals assessed in this study accumulated in fish directly from the water and planktonic life in the Indus River in the study area. In this study, higher concentrations of salinity, sulfates, phenol and heavy metals were the driving force which decreased the abundance of phytoplankton and zooplankton, and their FA profile. The phenol, sulfate, total dissolved solids (TDS), and TS values clearly indicated difference in their concentration at HP and LP sites. The level of salinity at HP was very close to that of brackish waters. The higher concentration of total TDS and TS at HP may be due to high turbidity. The presence of different metals in freshwater ecosystems varied with the physico-chemical factors of the corresponding ecosystem, particularly the pH and redox state. Reference [41] reported that the decrease in pH at high river discharges may release metals from complexes in the river and streams, which may be toxic to the aquatic fauna and flora in the ecosystem.

The levels of heavy metals in the water samples collected from HP passed the upper limits recommended by reference [37]. The heavy metals level in the water samples and in the muscles of *H. molitrix, C. idella*, and *C. carpio*, and planktonic biomass collected from HP. The bioaccumulation of heavy metals is known to influence the FA profile of fish. Reference [42,43] mentioned that metals stimulate cellular synthesis and metabolism of FA through β-oxidation, while pharmaceutical products act as peroxisomal proliferators [43,44,45]. Very limited information is available about the influence of heavy metals on Proliferation Activate Receptors (PPARs) expression and the transcription factors of FA metabolism in fish [46]. Elements in these fish species captured from HP and LP sites were accumulated by bio-concentration, and through food and water [16,37]. The increased concentration of heavy metals along with salinity and phenols at HP and LP sites compared to N P site probably are major factors which caused physiological variation in the food web and disturb the biosynthesis of FAs in *H. molitrix, C. idella*, and *C. carpio* [16,38]. The concentration of many heavy metals decreased in higher trophic levels in the food web [45]. In this study, similar results were obtained for most of the heavy metals, except for Hg [47,48]. This was particularly so in the higher trophic levels, and ultimately affected terrestrial ecosystems through fish [16,38].

PUFAs enter at the first trophic level of the food chain via FA synthesis in periphyton. Reference [49] has mentioned that light causes multiple effects on periphyton lipid metabolism and FA profiles. In general, higher light intensity normally causes oxidative damage to PUFA. In addition to the contamination, low light intensity and poor water quality at HP and LP sites influenced the abundance of periphyton producing high quality FA, thereby affecting PUFAs. The movement of FAs from periphyton to the fish level was found to be increasing with the pH at HP and LP sites. Thus, alkaline pH stress promoted an accumulation of TAG (Triacylglycerols) and a proportionally decrease in membrane lipids [50] In this research work, the changes in physio-chemical factors influenced the production of lipids in the planktonic life at HP and LP sites. The current findings seem to agree with the results of reference [51]. They had mentioned that phytoplanktonic abundance and their diversity were affected by eutrophication, which influence the FAs production due to interspecific variations in periphyton FA levels. However, there remains very little information on the molecular mechanisms involved in these abiotic environmental stressors.

The concentration of EPA, DHA and PUFAs was greater in the microalgae at LP compared to HP, which may due to the higher biomass of microalgae. The increase in microalgae growth is promoted by the higher concentration of nutrients, which might have promoted the synthesis of EPA [31,52]. Reference [53] reported that fluctuations in nutrient availability in the food chain affect on FAs profiles of periphyton. The fluctuations in FA profiles in the trophic levels of the aquatic food chain are probably due variations in the periphyton community composition. Our results of increases in the percentage of EPA were not in line with the results of reference [54]. Total PUFA and PUFA:SAFA ratios were reduced in periphyton and zoobenthos with increase in pollution at the HP site compared to the NP site. The level of PUFA in zoobenthos relies on various biotic and abiotic factors [50] such as food types and levels of contamination [31]. Reference [55] reported that increases in the concentration of Cd decreased the production FA profiles in *Chlorella vulgaris*. However, more Cd accumulated under N stress, which reduced the production of triglycerols in algae. DHA is necessary for the good growth of these freshwater fish species in aquatic ecosystems. The low level of DHA in planktonic food may affect the growth and development of different organs in freshwater fish species [31,56]. Here the reduction in the level of DHA was detrimental to the fatty acid profiles of *H. molitrix, C. idella*, and *C. carpio* from HP sites. The accumulation of PUFA in zoobenthos depends on various biotic and abiotic factors [52,57], food types [58] and pollution levels [37].

Fish are considered to be the best source of animal protein, globally. However, deterioration in their quality and losses in FAs cannot be recouped. Differences were non-significant for the FA profile in *C. carpio* sampled from HP and LP sites compared to NP sites, which exhibit an identical response to the chemical pollutants. The maximum percentage of SFAs in *C. carpio* was recorded in the fish procured from HP. Fish with a high concentration of SFAs need more energy for their movement and to search for food [59]. The SFAs C8:0, C12:0 and C20:0 were not recorded for *H. molitrix, C. idella*, and *C. carpio* captured from HP sites. The higher levels of heavy metals at HP and LP sites in the river adversely affected the synthesis of FAs in the three fish species. The higher SFA levels are probably due to de novo synthesis within these fish species. The heavy metals accumulate towards the bottom of the river, and *C. carpio* feeds on a variety of benthic organisms and macrophytes, thereby exposing it to high proportions of heavy metals [60]. The total MUFA concentrations recorded were supported by the findings of reference [61].

A significant lower percentage of PUFAs was noticed in *H. molitrix, C. idella*, and *C. carpio* from HP and LP. However, H. molitrix exhibited higher levels of ω-3 FAs and a large loss of ω-6 fatty acids. Eicosapentaenoic acid (C20:5n3) was not recorded in *C. idella* and *C. carpio* from HP. The concentrations of EP and C20: 5n3 were lower than those of menhaden oil. Identical results were reported by reference [62]. Linoleic acid, eicosadienoic acid and docosapentaenoic acid were not detected in fish procured from HP. Eicosapentaenoic acid was detected only in the muscle samples of H. molitrix from HP. The reduction in the production of PUFAs in *H. molitrix*, *C. idella*, and *C. carpio* from HP and LP may be due to increased levels of metals in the water at these locations in the river [63]. The zooplankton is a source of EPA and DHA for fish in the aquatic ecosystem [26]. The alterations in the food web, linked with an increase in environmental stress in freshwater ecosystems, invasive species, and habitat deterioration, may cause a significant variation in pollutant and lipid trophic transfer [26,64]. *C. idella* captured from HP and LP sites surprisingly responded to the general environment for FA profiles, although they feed on aquatic vegetation. We did not work on the FA profile of aquatic vegetation, and suspect that the alteration in the FA profile was due to an increased water pollution. The higher metal concentration might have affected the FA profile of the aquatic vegetation. This aspect may be verified in future studies. The alterations in the food web, linked with an increase in environmental stress in freshwater ecosystems, invasive species, and habitat deterioration, may cause a significant variation in pollutant and lipid trophic transfer [16]. The variations in FA and heavy metals trophic transfers in the food chain can provide insights into ecological functioning and the fallout of environmental stressors on the FA profile of different organisms in freshwater food webs.

## 5. Conclusions

Lipids play a significant role in the bioaccumulation of lipophilic pollutants in freshwater fish. The increase of heavy metals in the waters of the Indus River has produced trophic transfers to periphyton, zoobenthos, and fish in highly polluted (HP) and less polluted (LP) sites. Polyunsaturated Fatty Acids (PUFAs) level was greater in periphyton and zoobenthos biomass from non-polluted (NP) sites, compared to HP and LP sites. Fatty acids in the fish muscles were affected by the level of contamination due to the alterations in the food web, linked with an increase in environmental stress, invasive species, and habitat deterioration. It has been inferred that abiotic factors and chemical pollutants induced the trophic transfer in the food, and ultimately the loss of essential fatty acids (FAs) in fish meat. The variations in FA and heavy metals trophic transfers in the food chain can provide insights into ecological functioning and the fallout of environmental stressors on the FA profile of different organisms in freshwater food webs.

It is proposed that FAs may be used to evaluate trophic relationships among water, planktonic life forms, and fish in the food web in order to provide information to consumers about the safety of fish meat. Thus, the variation in FA profiles may be used as a biomarker to assess the status of the health of the ecosystem, and possibly to identify the causes of decreases in the abundance of fish populations.

## Figures and Tables

**Table 1:** Mean Physico-Chemical parameters and metal concentrations (± SE) at different sampling locations of Indus River.

Water Quality Characteristics	HP Site	LP Site	NP Site	Permissible Limits
pH	12.1 ± 0.36 a	8.5 ± 0.12 b	8.1 ± 0.08 b	D: 6.5–8.5, P: **
BOD (mg/L)	81.2 ± 1.10 a	48.8 ± 0.41 b	36.7 ± 0.77 c	†D: 30 mg/L, P: **
COD (mg/L)	195.8 ± 1.16 a	71.2 ± 0.90 b	65.5 ± 0.58 c	†D: 250 mg/L, P: **
TDS (mg/L)	2444.5 ± 8.41 a	1319.8 ±10.62 b	340.3 ± 7.24 c	D: 500 mg/L, P: 2000 mg/L
TSS (mg/L)	329.6 ± 6.41 a	218.6 ± 5.15 b	190.6 ± 4.24 c	D: 100mg/L, P: **
Salinity (mg/L)	1951.2 ±18.31 a	458.5 ± 7.22 b	242.3± 4.90 b	P: <100 mg/L
Conductivity µS/cm)	4.1 ± 0.22 a	1.55 ± 0.11 b	0.42 ± 0.051 c	D:650 µS/cm, P: 1055 µS/cm
Phenols (mg/L)	2.49 ± 0.18 a	0.84 ± 0.04 b	0.21 ± 0.01 c	D: 0.001 mg/L, P: 0.002 mg/L
Sulfates (mg/L)	452.3 ± 7.62 a	341.21± 0.08 b	97.4 ± 3.90 c	D: 0.001 mg/L, P: 0.002 mg/L
**Heavy Metal Contamination**				
Sn (mg/L)	0.54 ± 0.02 a	0.03 ± 0.0 b	0.01 ± 0.0 b	D: 0.01 mg/L, P: **
Cr (mg/L)	0.72 ± 0.03 a	0.36 ± 0.02 b	0.05 ± 0.00 c	D: 0.05 mg/L, P: **
Pb (mg/L)	3.02 ± 0.07 a	0.21 ± 0.02 b	0.14 ± 0.01 c	D: 0.05 mg/L, P: **
Zn (mg/L)	0.56 ± 0.02 a	0.251 ± 0.03 b	0.05 ±0.00 a	D: 5 mg/L, P: 15 mg/L
Mn (mg/L)	2.81 ± 0.12 a	2.05 ± 0.06 a	0.41 ± 0.01 c	D: 0.1 mg/L, P: 0.3 mg/L
Cu (mg/L)	2.05 ± 0.05 a	0.99 ± 0.11 b	0.08 ± 0.00 c	D: 0.05 mg/L, P: 1.5 mg/L
Cd (mg/L)	0.29 ± 0.02 a	0.03 ± 0.00 b	0.00 ± 0.00 b	D: 0.01 mg/L, P: **
Hg (mg/L0	1.51 ± 0.04 b	0.05 ± 0.01 c	< 0.001	D: 0.001 mg/L, P: **

BOD: Biological oxygen demand, COD: Chemical oxygen demand. TDS: Total dissolved solids, TSS: Total suspended solids. Different letters (a, b, c) in the same row represent significant (*p* < 0.05) differences. D; Desirable limits. P; Permissible limits. †; Effluent inland surface water quality standards. ** No relaxation.

**Table 2:** Heavy metal concentrations (mg/kg) in the biomass of periphyton, zoobenthos and in the muscle of fish species from different sampling locations of the Indus River.

Parameter	HP Site	LP Site	NP Site
**Periphyton**			
Sn	17.61 ± 0.90 a	11.34 ± 0.77 b	4.88 ± 0.61 c
Cr	6.10 ± 0.70 a	1.98 ± 0.55 b	0.15 ± 0.23 c
P b	1.68 ± 0.67 a	0.41 ± 0.10 b	0.15 ± 0.03 c
Zn	14.46 ± 1.35 b	23.11 ± 1.66 a	8.19 ± 0.1.05 c
Mn	20.66 ± 1.41 a	11.44 ± 1.33 a	5.90 ± 0.88 c
Cu	18.43 ± 1.44 a	6.97 ± 0.92 b	4.02 ± 0.55 c
Cd	2.41 ± 0.31 a	0.62 ± 0.12 b	0.16 ± 0.02 c
Hg	3.78 ± 0.40 b	1.70 ± 0.18 c	0.17 ± 0.00 c
**Zoobenthos**			
Sn	3.43 ± 0.31 a	2.90 ± 0.70 b	1.11 ± 0.40 c
Cr	2.80 ± 0.42 a	1.22 ± 0.18 b	0.26 ± 0.03 c
P b	1.21 ± 0.05 a	0.38 ± 0.01 b	0.15 ± 0.02 c
Zn	6.94 ± 0.77 b	8.06 ± 1.0 a	3.80 ±0.22 c
Mn	3.91 ± 0.48 a	1.30 ± 0.26 b	0.92 ± 0.01 c
Cu	6.02 ± 0.72 a	1.40 ± 0.21 b	0.33 ± 0.03 c
Cd	1.99 ± 0.18 a	0.89 ± 0.02 b	0.36 ± 0.07 c
Hg	1.92 ± 0.31 b	0.86 ± 0.18 c	0.09 ± 0.00 c
***Hypophthalmichthys molitrix***			
Sn	1.95 ± 0.41 a	1.32 ± 0.36 b	0.67± 0.16 c
Cr	3.01 ± 0.60 a	1.71 ± 0.22 b	0.82 ± 0.15 c
P b	0.81 ± 0.16 a	0.49 ± 0.08 b	0.21 ± 0.01 c
Zn	5.67 ± 0.67 a	3.62 ± 0.41 b	1.62 ± 0.33 c
Mn	2.70 ± 0.41 a	1.92 ± 0.38 b	0.79 ± 0.08 c
Cu	4.93 ± 0.62 a	1.08 ± 0.15 b	0.49 ± 0.05 c
Cd	1.41 ± 0.28 a	0.76 ± 0.12 b	0.35 ± 0.05 c
Hg	1.37 ± 0.22 a	0.87 ± 0.09 b	0.06 ± 0.00 c
***Ctenopharyngodon idella***			
Sn	1.81 ± 0.3 a	1.12 ± 0.18 b	0.61 ± 0.06 c
Cr	2.28 ± 0.27 a	1.57 ± 0.20 b	0.71 ± 0.15 c
P b	0.74 ± 0.10 a	0.49 ± 0.06 b	0.23 ± 0.01 c
Zn	5.62 ± 0.72 a	3.01 ± 0.47 b	1.52 ± 0.31 c
Mn	2.50 ± 0.38 a	1.62 ± 0.21 b	0.69 ± 0.17 c
Cu	4.70 ± 0.52 a	1.02 ± 0.20 b	0.42 ± 0.05 c
Cd	1.34 ± 0.31 a	0.70 ± 0.21 b	0.31 ± 0.06 c
Hg	1.41 ± 0.22 a	0.87 ± 0.16 b	0.09 ± 0.00 c
***Cyprinus carpio***			
Sn	2.41 ± 0.4 a	1.61 ± 0.17 b	0.72 ± 0.06 c
Cr	3.44 ± 0.40 a	1.90 ± 0.23 b	0.95 ± 0.16 c
P b	0.98 ± 0.12 a	0.69± 0.07 b	0.40 ± 0.03 c
Zn	6.75 ± 0.88 a	3.96 ± 0.60 b	1.88 ± 0.44 c
Mn	2.90 ± 0.40 a	1.80 ± 0.24 b	0.77 ± 0.18 c
Cu	5.48 ± 0.80 a	1.57 ± 0.28 b	0.58 ± 0.02 c
Cd	1.62 ± 0.40 a	0.88 ± 0.16 b	0.45 ± 0.05 c
Hg	1.69 ± 0.21 a	0.92 ± 0.18 b	0.22 ± 0.01 c

Values (Mean ± SE) are averages of five samples analyzed in triplicate. Different letters (a, b, c) in the same row represent significant (*p* < 0.05) differences.

**Table 3:** Fatty acids (% ±SE) in periphyton and zoobenthos from three sampling sites at different pollution levels in the Indus River.

Pytoperiphyton			
Fatty Acids	HP Site	LP Site	NP Site
**SFAs**			
C8:0	----------	----------	----------
C10:0	3.33 ± 0.11 c	6.78 ± 0.88 b	8.12 ± 0.98 a
C12:0	----------	---------	---------
C14:0	1.67 ± 0.08 c	4.41 ± 0.16 b	5.69 ± 0.77 a
C16:0	16.69 ± 2.77 c	19.63 ± 2.55 b	21.89 ± 2.44 a
C18:0	12.47 ± 1.63 c	13.12 ± 1.20 b	15.76 ± 2.89 a
C20:0	----------	---------	0.67 ± 0.11
**MUFAs**			
C16:1(n-7)	---------	---------	---------
C16:1(n-9)	---------	---------	---------
C18:1(n-7)	---------	----------	1.99± 0.16 a
C18:1(n-9)	---------	---------	0.97 ± 0.11
C20:1(n-9)	---------	---------	---------
C22:1(n-9)	3.11± 0.13 c	5.12 ± 0.34 b	7.89 ± 0.25 a
**PUFAs**			
C18:2(n-6)	0.43 ± 0.04 c	0.66 ± 0.19 b	1.15 ± 0.22 a
C18:3(n-3)	8.31 ± 0.70 b	9.11 ± 0.90 a	9.44 ± 0.88 a
C18:4(n-3)	---------	---------	---------
C20:2(n-6)	---------	-----------	2.24 ± 0.05 a
C20:4(n-6)	6.11 ± 0.55 c	8.02 ± 0.71 b	9.98 ± 0.41 a
C20:5(n-6)	---------	4.11 ± 0.22 b	5.23 ± 0.28 a
C20:5(n-3)	6.78 ± 0.90 a	5.97 ± 0.60 a	4.66 ± 0.70 c
C22:4(n-6)	---------	---------	---------
C22:5(n-6)	-----------	----------	2.79 ± 0.24 a
C22:5(n-3)	6.57 ± 0.41 c	7.33 ± 0.70 b	8.95± 0.66 a
C22:6(n-3)	5.44 ± 0.25 a	4.76 ± 0.41 a	3.77 ± 0.33 b
**Zoobenthos**			
**Fatty acids**	**Highly polluted water**	**Less polluted water**	**Non-polluted site**
**SFAs**			
C8:0	----------	----------	----------
C10:0	1.44 ± 0.23 c	4.11 ± 0.71 b	6.32 ± 0.71 a
C12:0	----------	---------	---------
C14:0	0.99 ± 0.08 c	2.98 ± 0.27 b	4.22 ± 0.55 a
C16:0	13.22 ± 1.66 c	15.38 ± 2.80 b	18.45 ± 2.88 a
C18:0	7.23 ± 1.66 b	10.45 ± 1.76 b	14.77 ± 2.18 a
C20:0	----------	---------	0.1 ± 0.11
**MUFAs**			
C16:1(n-7)	---------	---------	---------
C16:1(n-9)	---------	---------	---------
C18:1(n-7)	---------	---------	1.66 ± 0.07 a
C18:1(n-9)	---------	---------	0.77 ± 0.08
C20:1(n-9)	---------	---------	---------
C22:1(n-9)	1.72 ± 0.14 c	3.77 ± 0.53 b	4.99 ± 0.66 a
**PUFAs**			
C18:2(n-6)	0.20 ± 0.02 c	0.41 ± 0.28 b	0.81 ± 0.30 a
C18:3(n-3)	4.10 ± 0.7 c	6.22 ± 0.41 b	7.69 ± 0.44 a
C18:4(n-3)	---------	---------	---------
C20:2(n-6)	---------	-----------	2.89 ± 0.14
C20:4(n-6)	3.00 ± 0.49 c	4.77 ± 0.79 b	6.67 ± 0.51 a
C20:5(n-6)	---------	1.90 ± 0.27 b	3.69 ± 0.47 a
C20:5(n-3)	4.89 ± 0.71 a	3.81 ± 0.20 a	2.44 ± 0.22 b
C22:4(n-6)	---------	---------	---------
C22:5(n-6)	0.60 ± 0.09 b	1.00 ± 0.11 b	1.89 ± 0.47 a
C22:5(n-3)	4.12 ± 0.70 c	6.22 ± 0.60 b	7.52 ± 0.70 a
C22:6(n-3)	4.78 ± 0.31 a	3.83 ± 0.54 a	2.77 ± 0.40 c

SFAs: Saturated fatty acids; MUFAs; Monounsaturated fatty acids; PUFAs: Polyunsaturated fatty acids; Values (Mean ± SE) are averages of five samples for each fish species analyzed in triplicate. Different letters (a, b, c) in the same row represent significant (*p* < 0.05) differences.

**Table 4:** Fatty acid profile % (±SE) of fish muscle from three sites at different pollution levels.

Less Polluted Site (LP)			
Fatty Acids	*H. molitrix*	*C. idella*	*C. carpio*
**SFAs**			
C8:0	--------	----------	-------------
C10:0	1.02 ± 0.07 a	0.41 ± 0.01a b	1.44 ± 0.03 a
C12:0	0.01 ± 0.00 a b	0.01± 0.03 a	0.74 ± 0.06 a
C14:0	1.54 ± 0.04 a	1.37 ± 0.03 a	0.53 ± 0.02 b
C16:0	41.22 ± 2.70 a	37.66 ± 2.61 a	43.98 ± 2.14 a b
C18:0	0.47 ± 0.05 b	24.66 ± 3.40 a b	29.51 ± 2.67 b
C20:0	-----------	------------	1.45 ± 0.03
**MUFAs**			
C16:1(n-7)	0.44 ± 0.04 a	----------	----------
C16:1(n-9)	0.71 ± 0.10 a b	0.79 ± 0.02 b	0.29 ± 0.01 a b
C18:1(n-7)	0.14 ± 0.08 b	0.08 ± 0.01a b	0.40 ± 0.04 b
C18:1(n-9)	3.95 ± 0.33 b	4.79 ± 0.04 a	0.11 ± 0.02 a b
C20:1(n-9)	0.77 ± 0.03	----------	-----------
C22:1(n-9)	0.90 ± 0.06 b	0.41 ± 0.03 a	0.32 ± 0.04 a b
**PUFAs**			
C18:2(n-6)	0.70 ± 0.06 a b	1.29 ± 0.3 a	0.41 ± 0.07 a b
C18:3(n-3)	12.66 ± 0.79 a	3.82 ± 0.04 a	0.003 ± 0.00 a b
C18:4(n-3)	3.24 ± 0.19 a	0.44 ± 0.05 a	2.60 ± 0.02 a b
C20:2(n-6)	-----------	---------------	---------------
C20:4(n-6)	0.87 ± 0.09 a	0.39 ± 0.02 a	0.82 ± 0.01 a
C20:5(n-6)	12.06 ± 0.54 a	8.02 ± 0.24 a	9.16 ± 0.42 a
C20:5(n-3)	2.42± 0.30 b	0.37 ± 0.18 c	3.61 ± 0.22 a
C22:4(n-6)	---------	-----------	----------
C22:5(n-6)	16.77 ± 0.66 a	4.70 ± 0.12 b	5.44 ± 0.22 b
C22:5(n-3)	1.17± 0.10 b	0.02 ± 0.00 c	1.88 ± 0.06 b
C22:6(n-3)	4.02 ± 0.33 a b	2.94 ± 0.22 a	3.71 ± 0.14 a b
**Highly polluted site (HP)**			
**Fatty acids**	***H. moiltrix***	***C. idella***	***C. carpio***
**SFAs**			
C8:0	----------	----------	----------
C10:0	0.18 ± 0.03 c	1.97 ± 0.11 b	6.93 ± 0.77 a
C12:0	----------	---------	---------
C14:0	0.44 ± 0.05 c	1.83 ± 0.21 b	2.65 ± 0.22 a
C16:0	34.25 ± 4.66 c	44.25 ±5.26 b	50.12 ± 4.77 a
C18:0	27.18 ± 3.16 b	35.69 ± 3.75 a	12.24 ± 1.77 c
C20:0	----------	0.25 ± 0.01	---------
**MUFAs**			
C16:1(n-7)	---------	---------	---------
C16:1(n-9)	---------	---------	---------
C18:1(n-7)	5.22 ± 0.52	---------	---------
C18:1(n-9)	0.62 ± 0.06 a	---------	0.49 ± 0.01 a
C20:1(n-9)	---------	---------	---------
C22:1(n-9)	0.32 ± 0.02 c	5.61 ± 0.17 a	4.11 ± 0.22 b
**PUFAs**			
C18:2(n-6)	---------	---------	---------
C18:3(n-3)	16.55 ± 0.54 a	3.66 ± 0.32 b	0.24 ± 0.01 c
C18:4(n-3)	---------	---------	---------
C20:2(n-6)	---------	0.87 ± 0.11 a	0.59 ± 0.02 b
C20:4(n-6)	4.72 ± 0.33 b	7.42 ± 0.22 a	3.13 ± 0.07 c
C20:5(n-6)	---------	---------	0.92 ± 0.11
C20:5(n-3)	0.68± 0.07	---------	---------
C22:4(n-6)	---------	---------	---------
C22:5(n-6)	0.52 ± 0.11 b	0.17 ± 0.01 c	0.88 ± 0.11 a
C22:5(n-3)	6.12 ± 0.44 a	4.12 ± 0.22 b	---------
C22:6(n-3)	3.62 ± 0.11 a	0.21 ± 0.01 c	1.76 ± 0.22 b
**Non-polluted site (NP)**			
**Fatty acids**	***H. moiltrix***	***C. idella***	***C. carpio***
**SFAs**			
C8:0	0.80 ± 0.05 a	0.01 ± 0.01 a	----------
C10:0	0.31 ± 0.00 b	3.15 ± 0.03 a	3.67 ± 0.08 a
C12:0	1.21± 0.15 c	3.10 ± 0.40 a	2.16 ± 0.05 b
C14:0	0.45 ± 0.01 c	4.68 ± 0.55 b	7.79± 0.44 a
C16:0	17.10 ± 0.61 a	14.78 ± 1.12 b	12.42 ± 0.55 b
C18:0	6.01 ± 0.41 a	3.80 ± 0.25 c	8.90 ± 0.75 a
C20:0	1.42 ± 0.17 a	0.32 ± 0.01 b	0.38 ± 0.01 b
**MUFAs**			
C16:1(n-7)	--------	--------	0.72 ± 0.04
C16:1(n-9)	4.75 ± 0.16 a	2.41 ± 0.04 b	2.77 ± 0.05 b
C18:1(n-7)	4.81 ± 0.45 a	3.60 ± 0.02 b	1.81 ± 0.11 b
C18:1(n-9)	13.12 ± 0.82 a	12.87 ± 0.66 a	10.94 ± 0.42 a
C20:1(n-9)	1.44 ± 0.07 b	4.14 ± 0.51 a	3.88 ± 0.22 a
C22:1(n-9)	0.01 ± 0.00 c	0.61 ± 0.01 a	0.70 ± 0.02 a
**PUFAs**			
C18:2(n-6)	4.07 ± 0.06 a	3.42 ± 0.11 a	0.01 ± 0.00 b
C18:3(n-3)	3.44 ± 0.23 b	3.22 ± 0.22 b	5.94 ± 0.41 a
C18:4(n-3)	2.41 ± 0.11 a	2.05 ± 0.07 a	0.71 ± 0.02 b
C20:2(n-6)	0.91 ± 0.02 a	0.88 ± 0.01 a	0.57 ± 0.02 b
C20:4(n-6)	14.26 ± 0.87 a	12.14 ± 0.28 b	9.87 ± 0.71 c
C20:5(n-6)	6.44 ± 0.28 a	4.12 ± 0.20 b	0.21 ± 0.01 c
C20:5(n-3)	0.40 ± 0.01 c	5.06 ± 0.31 b	8.23 ± 0.60 a
C22:4(n-6)	1.21 ± 0.02 a	0.60 ± 0.00 b	0.50 ± 0.00 b
C22:5(n-6)	3.44 ± 0.32 b	4.02 ± 0.09 b	6.68 ± 0.25 a
C22:5(n-3)	5.05 ± 0.33 a	3.60 ± 0.22 b	2.39 ± 0.04 c

SFAs: Saturated fatty acids; MUFAs; Monounsaturated fatty acids; PFAs: Polyunsaturated fatty acids; Values (Mean ± SE) are averages of five samples for each fish species analyzed in triplicate. Different letters (a, b, c) in the same row represent significant (*p* < 0.05) differences.

**Table 5:** Correlation matrix for metal concentrations with total fatty acids in periphyton, zoobenthos, and fish in three sites at different pollution levels.

Metals	HP-PP	LP-PP	NP-PP	HP-ZB	LP-ZB	NP-ZB	HP-HM	LP-HM	NP-HM	HP-GC	LP-GC	NP-GC	HP-CP	LP-CP	NP-CP
Sn	−0.24 *	−0.13	0.07	−0.37 *	−0.28 *	0.05	−0.46 **	−0.24 *	0.06	−0.30 *	−0.31 *	0.08	−0.60 **	−0.27 *	0.05
Cr	−0.56 **	−0.33 *	0.10	−0.58 **	−0.33 *	0.02	−0.33 *	−0.24 *	0.04	−0.58 **	−0.31 *	−0.07	−0.54 **	−0.28 *	−0.11
P b	−0.37 *	−0.26 *	−0.06	−0.58 **	−0.28 *	−0.05	−0.44 **	−0.25 *	0.03	−0.40 **	−0.22 *	0.01	−0.560 **	−0.27 *	−0.01
Zn	−0.51 **	−0.23 *	0.03	−0.53 **	−0.25 *	−0.07	−0.34 *	−0.23 *	0.01	−0.28 *	−0.27 *	−0.03	−0.57 **	−0.24 *	0.09
Mn	−0.60 **	−0.28 *	0.02	0.56 **	−0.27 *	−0.09	−0.48 **	−0.24 *	−0.02	−0.64 **	−0.26 *	0.04	−0.57 **	0.28 *	−0.05
Cu	−0.60 **	−0.27 *	0.05	−0.57 **	−0.44 **	−0.08	−0.53 **	−0.25 *	0.03	−0.37 *	−0.28 *	−0.06	−0.57 **	−0.29 *	0.04
Cd	−0.27 *	−0.24 *	0.10	−0.33 *	−0.25 *	−0.07	−0.41 *	−0.27 *	−0.12	−0.42 **	−0.28 *	0.02	−0.51 **	−0.25 *	−0.05
Hg	−0.34 *	−0.22 *	0.01	−0.27 *	0.12	0.01	−0.28 *	−0.22 *	0.004	−0.35 *	−0.27 *	−0.01	−0.48 **	−0.26 *	0.003

*significant at 0.05 level; ** significant at 0.01 level; HP: highly polluted site; LP: low polluted site; NP: Non-polluted site; PP: periphyton; ZB: zoobenthos; HM: *H. moiltrix*; CI: *C. idella*; CP: *C. carpio*.
